# Bioactive Glass and Glass-Ceramic Scaffolds for Bone Tissue Engineering

**DOI:** 10.3390/ma3073867

**Published:** 2010-07-06

**Authors:** Lutz-Christian Gerhardt, Aldo R. Boccaccini

**Affiliations:** 1 Department of Materials, Imperial College London, Prince Consort Road, London SW7 2BP, UK; 2 Institute of Biomaterials, University of Erlangen-Nuremberg, 91058 Erlangen, Germany

**Keywords:** bioactive glasses, glass-ceramics, melt-derived glasses, scaffolds, bone, tissue engineering, composites, ion release, osteogenesis, angiogenesis

## Abstract

Traditionally, bioactive glasses have been used to fill and restore bone defects. More recently, this category of biomaterials has become an emerging research field for bone tissue engineering applications. Here, we review and discuss current knowledge on porous bone tissue engineering scaffolds on the basis of melt-derived bioactive silicate glass compositions and relevant composite structures. Starting with an excerpt on the history of bioactive glasses, as well as on fundamental requirements for bone tissue engineering scaffolds, a detailed overview on recent developments of bioactive glass and glass-ceramic scaffolds will be given, including a summary of common fabrication methods and a discussion on the microstructural-mechanical properties of scaffolds in relation to human bone (structure-property and structure-function relationship). In addition, ion release effects of bioactive glasses concerning osteogenic and angiogenic responses are addressed. Finally, areas of future research are highlighted in this review.

## 1. Introduction

Tissue engineering (TE) and regenerative medicine aim to restore diseased or damaged tissue using combinations of functional cells and biodegradable scaffolds made from engineered biomaterials [1,2]. Some of the most promising biomaterials for application in bone tissue engineering are bioceramics such as hydroxyapatite (HA), calcium phosphates, bioactive glasses and related composite materials combining bioactive inorganic materials with biodegradable polymers [3,4]. Bioactive inorganic materials are capable of reacting with physiological fluids forming tenacious bonds to bone through the formation of bone-like hydroxyapatite layers leading to effective biological interaction and fixation of bone tissue with the material surface [5,6]. Moreover, in the case of silicate bioactive glasses, such as 45S5 Bioglass^®^ [5], reactions on the material surface induce the release and exchange of critical concentrations of soluble Si, Ca, P and Na ions, which can lead to favorable intracellular and extracellular responses promoting rapid bone formation [7,8,9,10,11].

In 1969, Hench and colleagues discovered that rat bone can bond chemically to certain silicate-based glass compositions [8,9]. This group of glasses was later termed “bioactive”, being “a material that elicits a specific biological response at the material surface which results in the formation of a bond between the tissues and the materials” [9,12]. Hench [8] has recently published the history leading to the development of bioactive glass (BG) focusing on the breakthrough discovery of the classical 45S5 Bioglass^®^ composition to successful clinical applications and tissue engineering. This oldest BG composition consists of a silicate network (45 wt % SiO_2_) incorporating 24.5 wt % Na_2_O, 24.5 wt % CaO and 6 wt % P_2_O_5_ as network modifiers. The high amounts of Na_2_O and CaO, as well as the relatively high CaO/P_2_O_5_ ratio make the glass surface highly reactive in physiological environments [11]. Other bioactive glass compositions developed over the years contain no sodium or have additional elements incorporated in the silicate network such as fluorine [13], magnesium [14,15], strontium [16,17,18], iron [19], silver [20,21,22,23], boron [24,25,26,27], potassium [28] or zinc [29,30].

**Figure 1:**
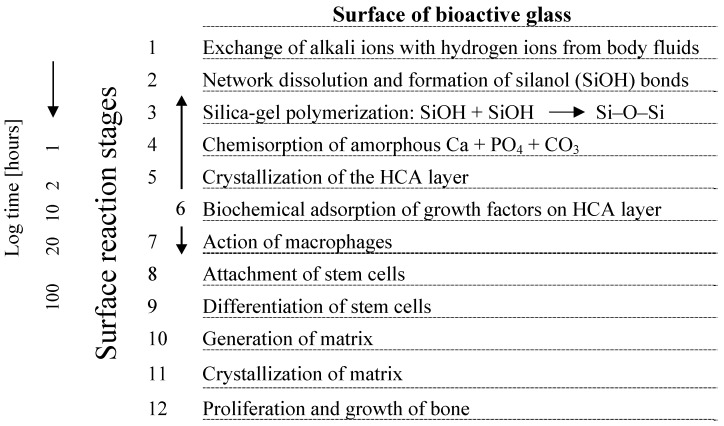
Sequence of interfacial reactions involved in forming a bond between bone and a bioactive glass (modified after reference [5]).

Fabrication techniques for bioactive glasses include both traditional melting methods and sol-gel techniques [1,3,4,10,31,32,33], the latter are being highlighted elsewhere [34] and are not covered in this review. The typical feature common to all bioactive glasses, being melt or sol-gel derived, is the ability to interact with living tissue, in particular forming strong bonds to bone (and in some cases soft tissue [35,36], a property commonly termed bioreactivity or bioactivity [1], as mentioned above. It is now widely accepted that for establishing bond with bone, such a biologically active apatite surface layer must form at the material/bone interface [1,8,11,12,37] (see also discussion in §4). Thus, the basis of the bone bonding property of bioactive glasses is the chemical reactivity in physiological body fluids (*in vitro* and *in vivo*) resulting in the formation of a hydroxycarbonate apatite (HCA) layer to which bone can bond. This bonding to living bone tissue occurs upon a sequence of reactions on the material surface [9] followed by cellular reactions [5], both of which are explained in detail elsewhere [1,5,9,11,12]. Briefly, the processes on the glass surface are characterized by ion leaching/exchange, dissolution of the glass network and precipitation and growth of a calcium-deficient carbonated apatite (HCA) surface layer, whereas cellular reactions include colonization, proliferation and differentiation of relevant (bone) cells [11,12] (Figure 1). In parallel to the chemical reactions on the material surface leading to bone bonding, recent studies have proven that ion dissolution and release from BG activate gene expression in osteo-genitor cells that give rise to enhanced bone regeneration (see §4.1).

The development of such a bioactive apatite layer is the common characteristic of all known inorganic materials used for bone replacement, orthopedic implants and bone tissue engineering scaffolds [1,38]. Early clinical applications of bioactive glasses were in the form of solid pieces for small bone replacement, *i.e.*, in middle ear surgery [1,8,11]. Later, other clinical applications of bioactive glasses were proposed, for example in periodontology or as coating on metallic orthopedic implants [5,8]. Historically, the main function of biomaterials and implants has been to replace diseased or damaged tissues. During the past three decades, however, the strategy in biomaterial research began to shift from developing biomaterials with a bio-inert tissue response to producing bioactive components that could elicit a controlled action and reaction in the physiological environment [1]. Since the late 1990’s and the beginning of the new millennium, great potential has been attributed to the application of bioactive glasses in tissue engineering and regenerative medicine [39,40,41,42,43]. Bone tissue engineering is one of the most exciting future clinical applications of bioactive glasses. Both micron-sized and recently nanoscale particles [23,44,45] are considered in this application field, which includes also the fabrication of composite materials, e.g., combination of biodegradable polymers and bioactive glass [38,46,47,48,49,50], as discussed in detail in §3.2. Bioactive silicate glasses exhibit several advantages in comparison to other bioactive ceramics, e.g., sintered hydroxyapatite, in tissue engineering applications. For example, it has been demonstrated that dissolution products from bioactive glasses up-regulate the expression of genes that control osteogenesis [7,51], which explains the higher rate of bone formation in comparison to other inorganic ceramics such as hydroxyapatite [52]. Further studies using 45S5 Bioglass^®^ particles have shown encouraging results regarding potential angiogenic effects of Bioglass^®^, *i.e.*, increased secretion of vascular endothelial growth factor (VEGF) and VEGF gene expression *in vitro*, as well as enhancement of vascularization *in vivo* [53,54,55,56] (see §4). In addition, the incorporation of particular ions into the silicate network, such as silver [20,21,22] and boron [26,27], has been investigated in order to develop antibacterial and antimicrobial materials. Bioactive glasses can also serve as vehicle for the local delivery of selected ions being able to control specific cell functions [30,57,58,59,60,61,62,63,64]. For example, mesoporous BG microspheres have demonstrated enhanced haemostatic activity, as well as reduced clot detection times and increased coagulation rates compared to nonporous microspheres [65]. The release of calcium ions is believed to be responsible for its haemostatic properties [65]. Moreover, ferromagnetic bioactive glasses and glass-ceramics containing magnetite are being currently developed for hyperthermia treatment of cancer [19,66,67,68,69].

Bioactive glass-ceramics belong to the group of Class A bioactive materials which are characterized by both osteoconduction (*i.e.*, growth of bone at the implant surface) and osteoinduction (*i.e.*, activation and recruitment of osteoprogenitor cells by the material itself stimulating bone growth on the surface of the material) [5,8,33,70]. Differences between Class A and B bioactive materials are discussed elsewhere [8,33,70]. As indicated above, the range of bioactive glasses exhibiting these attractive properties has been extended over the years, in terms of both chemical composition and morphology, as new preparation methods have become available. At this point, for completeness, it has to be mentioned that an early significant modification of bioactive glasses was the development of apatite/wollastonite (A/W) bioactive glass-ceramics [71,72]. A recent review summarizing research on Ca-Si-based ceramics is available [73].

Bone tissue engineering scaffolds are generally highly porous, 3-dimensional (3D) templates, exhibiting tailored porosity, pore size and controlled interconnectivity [37,74]. Several scaffold fabrication techniques, including foam replication methods, salt or sugar leaching, thermally induced phase separation, microsphere emulsification sintering, electrospinning to form nanofibrous structures, computer assisted rapid prototyping techniques [75,76], textile and foam coating methods [60,77,78], as well as biomimetic approaches [79,80] to optimize the structure, properties and mechanical integrity of scaffolds have been reported in the literature. Comprehensive reviews of the general state-of-the art in scaffold manufacturing and optimization are available [3,4,37,38,77,81]. The bio-mimicry of human bone, *i.e.*, the design and incorporation of nano-topographic features on the scaffold surface architecture, in order to mimic the nanostructure of natural bone, is also becoming a significant area of research in bone tissue engineering [10,82,83,84].

This review is organized in the following manner. In section 2, we discuss the essential design requirements for bone tissue engineering scaffolds. Section 3 provides a comprehensive summary of the main bioactive glass and glass-ceramic scaffold fabrication technologies, followed by a discussion of scaffold microstructures developed (e.g., porosity, pore structure, pore interconnectivity) and relevant structural-mechanical properties correlations (structure-function and structure-property relationships) in relation to human bone. Section 4 reviews the latest developments in the field of ion release effects on cell and tissue response to bioactive glass scaffolds, highlighting the effect of dissolution products from bioactive glasses in relation to osteogenesis and angiogenesis. Finally, in section 5, limitations of recently developed silicate scaffolds are discussed, and areas where further research is needed are identified. This review thus gives a complete overview on recent developments in the field of bioactive glasses for tissue engineering, focusing on melt-derived BG, and represents a literature update as well as an expansion of previously presented articles on the topic [34,73,77,81,85,86,87,88].

## 2. Basic Scaffold Requirements

**Figure 2:**
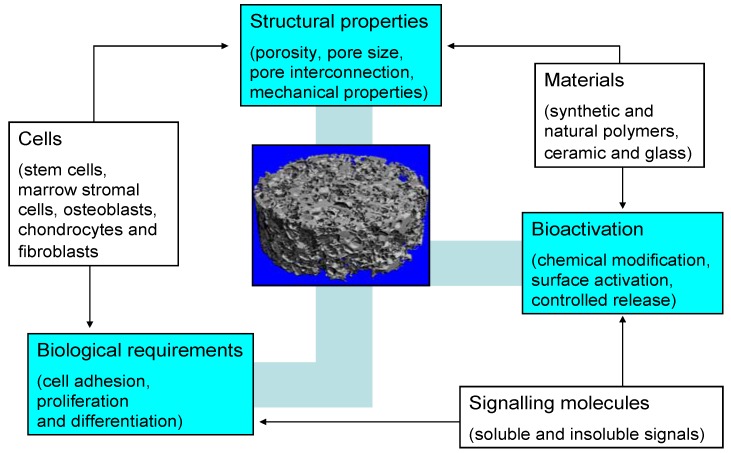
Schematic diagram of key factors involved in the design of optimal scaffolds for bone tissue engineering (modified after reference [3]).

The most important function of a bone TE scaffold is its role as template that allows cells to attach, proliferate, differentiate and organize into normal, healthy bone as the scaffold degrades. Figure 2 illustrates the most important factors involved in the design of TE scaffolds and their interdependencies, according to Guarino *et al.* [3]. Depending on the final application, scaffold requirements include matching the structural and mechanical properties with those of the recipient tissue and optimizing the microenvironment to foster cell integration, adhesion and growth, issues that have become known as structural and surface compatibility of biomaterials [89,90].

Scaffolds for bone tissue engineering are subjected to many interrelated biological and structural requirements which must be taken into consideration when selecting the suitable biomaterial for fabrication. Firstly, scaffolds need to encourage cell attachment, differentiation and proliferation which are cell functions highly dependent on substrate material properties. This is related to the property of osteoconductivity, which is important not only to avoid the formation of encapsulating tissue but also to induce a strong bond between the scaffold and host bone [5]. The rate of biodegradation *in vivo* is another criterion for selection of biomaterials for fabricating scaffolds, which should be tailored to match the rate of regeneration of new tissue. When considering biodegradable materials, it is also important to understand the time dependent variation of their mechanical properties and varying structural integrity since the mechanical strength of scaffolds has to be sufficient to provide mechanical stability in load-bearing sites during the period of new tissue formation.

Further requirements are related to the scaffold architecture. An ideal bone tissue scaffold should possess an interconnected porous structure, *i.e.*, it should be highly permeable (see §3.1), with porosity >90% and pore diameters in the range 10–500 μm for cell seeding, tissue ingrowth and vascularization, as well as for nutrient delivery and waste removal [37,38,74,91]. A particular design criterion of tissue engineering scaffolds is the mimicry and implementation of the bimodal porosity of cancellous bone tissue, which is an important factor for the effective scaffold vascularization and for bone ingrowth [92]. Microporosity (≈2–10 μm, <50 μm) is essential for immediate protein and cell adhesion, cell migration and osteointegration [13,74,92,93,94]. Higher pore sizes (>300 μm) are required for enhanced new bone formation, greater bone ingrowth and the formation of capillaries. Because of vascularization, pore size has been shown to affect the progression of osteogenesis. Small pores favored hypoxic conditions and induced osteochondral formation before osteogenesis, while large pores, that are well-vascularized, lead to direct osteogenesis (without preceding cartilage formation) [74]. However, higher scaffold porosity results in diminished mechanical properties thereby setting an upper functional limit for pore size and porosity. Thus, a balance must be reached depending on the repair, rate of remodeling and rate of degradation of the scaffold material [74], and the scaffold design has to consider an optimal porosity enabling sufficiently high permeability (*i.e.*, pore interconnectivity, see discussion in §3.1) for waste removal and nutrient supply and adequate stiffness and strength (see §3.1, Figure 7, Figure 9) to sustain the loads transmitted to the scaffold from the surrounding healthy bone [95]. Furthermore, scaffolds should be amenable to fabrication in complex or irregular shapes in order to match specific defect morphologies in bone of individual patients. Finally, material synthesis and fabrication of the scaffold should be suitable for sterilization as well as commercialization [8], *i.e.*, the technology of scaffold production must be scalable and cost-effective.

## 3. Silicate-Based Bioactive Glass Tissue Engineering Scaffolds

### 3.1. Bioactive Glass Based Glass-Ceramic Scaffolds

Glass-ceramics are partially crystallized glasses produced by heating the parent bioactive glass above its crystallization temperature, usually at about 610–630 °C [33,44,96,97]. In the case of glass-ceramics obtained by a sintering process, during the occurrence of crystallization and densification, the microstructure of the parent glass shrinks, porosity is reduced and the solid structure gains mechanical strength [70]. However, the brittleness and low fracture toughness remain a major impediment of these materials. The limited strength and low fracture toughness (*i.e.*, ability to resist fracture when a crack is present) of bioactive glasses has so far prevented their use for load-bearing implants [8,70,96,98], and thus the repair and regeneration of large bone defects at load-bearing anatomical sites (e.g., limbs) remains a clinical/orthopedic challenge [99,100]. Recent developments related to bone TE try to bridge this gap and overcome this problem by architectures and components carefully designed from comprehensive levels, *i.e.*, from the macro-, meso-, micrometer down to the nanometer scale [101], including both multifunctional bioactive glass composite structures (see §3.2) and advanced bioactive glass-ceramic scaffolds exhibiting oriented microstructures, controlled porosity and directional mechanical properties [99,102,103,104,105], as discussed in the following paragraphs. Most studies have investigated mainly the mechanical properties, *in vitro* and cell biological behavior of glass-ceramic scaffolds [13,14,15,30,43,52,94,95,97,99,102,103,104,105,106,107,108,109,110,111,112,113,114,115,116,117,118,119,120,121,122,123,124], as summarized in Table 1, and scaffolds with compressive strength [99,102] and elastic modulus values [99,105] in magnitudes far above that of cancellous bone and close to the lower limit of cortical bone have been realized.

**Table 1:** Overview on recent studies performed on silicate bioactive glass-ceramic scaffolds. The symbol § denotes fiber diameter.

Glass composition/system	Particle size of starting glass powder	Fabrication technique	Study
45S5	< 5 μm	Polymer foam replication	[95]
SiO_2_-CaO-CaF_2_-Na_2_O-K_2_O-P_2_O_5_-MgO	< 32 μm	Polymer foam replication	[13]
SiO_2_-P_2_O_5_-CaO-MgO-Na_2_O-K_2_O	< 30 µm	Polymer foam replication	[15,94]
SiO_2_-P_2_O_5_-CaO-MgO-Na_2_O-K_2_O	< 30 µm	Polymer foam replication	[108]
45S5	10–20 µm	Polymer foam replication	[118]
SiO_2_-Na_2_O-CaO-MgO	< 100 μm	Starch consolidation	[14]
SiO_2_-P_2_O_5_-B_2_O_3_-CaO-MgO-K_2_O-Na_2_O	75 μm^§^	Compaction and sintering of melt-spun fibers	[113]
SiO_2_-CaO-Na_2_O-K_2_O-P_2_O_5_-MgO-CaF_2_	< 106 µm	Polymer porogen bake-out	[102]
45S5	20–50 μm	Polymer foam replication	[97]
SiO_2_-Na_2_O-K_2_O-MgO-CaO-P_2_O_5_	255–325 μm	Slip casting	[107]
SiO_2_-Na_2_O-K_2_O-MgO-CaO-P_2_O_5_	< 5–10 μm	Polymer foam replication	[105]
SiO_2_-Na_2_O-K_2_O-MgO-CaO-P_2_O_5_	< 5 µm	Freeze casting	[99]
SiO_2_-CaO-K_2_O	< 106 µm	Polymer porogen burn-off	[106]
SiO_2_-TiO_2_-B_2_O_3_-P_2_O_5_-CaO-MgO-K_2_O-Na_2_O	75 μm^§^	Compaction and sintering of melt-spun fibers	[30]
45S5	45–90 μm	Polymer porogen bake-out	[119]
45S5	< 5 µm	Polymer foam replication	[103]
SiO_2_-Na_2_O-K_2_O-MgO-CaO-P_2_O_5_; 45S5	25–40 μm^§^	Densification and sintering of melt-spun fibers	[114]
45S5	≈ 5 μm	Polymer foam replication	[43]
45S5	5–10 μm	Polymer foam replication	[109]
45S5	≈ 10 μm	Polymer foam replication	[110]
SiO_2_-P_2_O_5_-CaO-MgO-Na_2_O-K_2_O	n.a.	Polymer burn-off, foam replication	[104]
45S5	< 5 µm	Polymer foam replication	[120]
SiO_2_-Na_2_O-CaO-P_2_O_5_-B_2_O_3_-TiO_2_	n.a.	Solution combustion	[52]
SiO_2_-Na_2_O-CaO-P_2_O_5_-B_2_O_3_-TiO_2_	n.a.	Solution combustion	[121]
SiO_2_-CaO-P_2_O_5_-Al_2_O_3_	8–30 μm^§^	Manual free-forming of melt- spun fibers	[122]
SiO_2_-CaO-Na_2_O-P_2_O_5_-K_2_O-MgO-B_2_O_3_	n.a.	Polymer foam replication	[123]
SiO_2_-CaO-Na_2_O-K_2_O-MgO-P_2_O_5_-B_2_O_3_	75 μm^§^	Densification and sintering of melt-spun fibers	[124]

Fu *et al.* [99] fabricated bioactive glass (13–93) scaffolds with oriented (*i.e.*, columnar and lamellar) microstructures and found that at an equivalent porosity of 55–60%, the columnar scaffolds had a compressive strength of 25 ± 3 MPa, compressive modulus of 1.2 GPa, and pore width of 90–110 µm, compared to values of 10 ± 2 MPa, 0.4 GPa, and 20–30 μm, respectively, for the lamellar scaffolds. The compressive strength of these columnar bioactive glass scaffolds is >1.5 times higher than the highest strength reported for trabecular bone (0.1–16 MPa, see Table 2). In addition, the cellular response of murine postosteoblasts/pre-osteocytes to columnar scaffolds indicated that these are most favorable for cell proliferation, migration, and mineralization (e.g., bone nodule formation, alkaline phosphatase activity). From the results in reference [99], the authors claimed that 13–93 bioactive glass scaffolds with columnar microstructure are promising candidate materials for the repair and regeneration of load-bearing bones *in vivo*. It is interesting to note in this regard that highly porous lamellar HA scaffolds (porosity ≈ 50–70%) fabricated by freeze casting exhibited 2.5–4 times higher compressive strengths (≈ 20–140 MPa) than conventional porous HA [101].

**Table 2:** Mechanical properties of human cancellous and cortical bone in comparison to dense bioactive glass 45S5 Bioglass^®^.

Material property	Trabecular bone	Cortical bone	Bioglass^®^ 45S5
Compressive strength [MPa]	0.1–16 [125,126]	130–200 [37,125]	500 [37]
Tensile strength [MPa]	n.a.	50–151 [37]	42 [70]
Compressive modulus [GPa]	0.12–1.1 [127,128]	11.5–17 [74]	n.a.
Young’s modulus [GPa]	0.05–0.5 [37,129]	7–30 [ 6,37,129]	35 [70]
Fracture toughness [MPa·m^1/2^]	n.a.	2–12 [37,70]	0.7–1.1 [130,131]

Multi-directional, anisotropic mechanical properties of scaffolds have been also reported by Baino *et al.* [102]. They prepared fluoroapatite containing glass-ceramic scaffolds and investigated their mechanical, structural and bioactive properties upon soaking in simulated body fluid (SBF). The scaffolds had interconnected macropores (porosity = 23.5–50%) and orthotropic mechanical properties, with compressive strength values in the range 20–150 MPa (Figure 7). Thick hydroxyapatite layers were formed on the surface of the scaffolds after 7 days of immersion in SBF, demonstrating the scaffold excellent bioactivity. Compressive strength values reported in refs. [99,102] are considerably higher than those found for bioactive glass-ceramic scaffolds with similar porosities (porosity = 54–73%), prepared by the foam replication technique [94]. The latter scaffolds formed from SiO_2_-P_2_O_5_-CaO-MgO-Na_2_O-K_2_O bioactive glass had a compressive strength of 1.3–5.4 MPa [94] (for comparison see Figure 7).

Ideally, the elastic modulus of the scaffold should be comparable to that of the tissue to be replaced in order to promote load transfer and minimize stress shielding, reducing the problems of bone resorption [132]. Stress shielding describes the mismatch in elastic moduli between biomaterial and the adjacent/surrounding bone. In case of large elastic mismatch, bone becomes “stress shielded”, which is undesirable since living bone must be under some tensile load stimuli to remain healthy. In the literature, depending on the measurement technique and parameters used, the source of bone and the structural variation in bone from the same source, a wide range of values has been reported for the compressive modulus of trabecular (0.12–1.1 GPa) and cortical bone (11.5–17 GPa) (Table 2 ).

**Figure 3:**
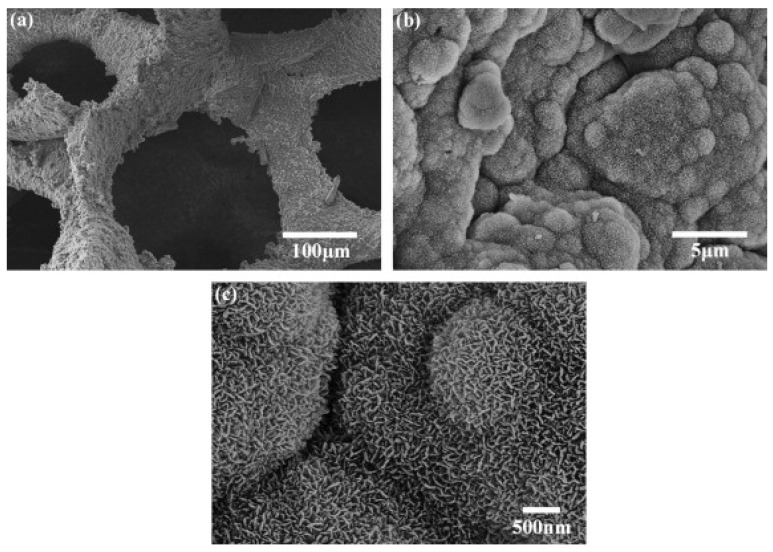
SEM images of the surface of a 13–93 glass  scaffold fabricated by a foam replication method, after immersion for 7 days in SBF: (a) lower magnification image; and (b, c) higher magnification image showing fine needle-like hydroxyapatite crystals [105]. Figure reprinted with permission of Elsevier.

**Figure 4:**
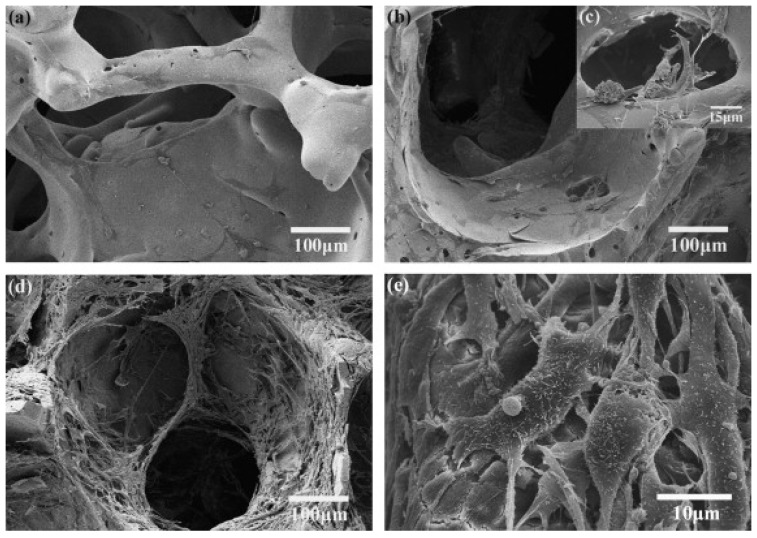
SEM images of 13–93 glass scaffolds seeded with MC3T3-E1 cells and cultured for: (a) 2 days; (b, c) 4 days; and (d, e) 6 days [105]. Reprinted with permission of Elsevier.

Fu *et al.* [105] reported for magnesium and potassium substituted bioactive glass-ceramic scaffolds (porosity = 85 ± 2%, pore size = 100–500 μm) a compressive strength of 11 ± 1 MPa and compressive modulus of 3.0 ± 0.5 GPa, which match the highest values reported for human trabecular bone (Table 2). Interestingly, these values are more than 10 times higher than compressive strengths reported for 45S5 Bioglass^®^ based scaffolds [43] of similar porosity and prepared by the same foam replication method. This finding confirms that glass composition and sintering parameters also affect the mechanical properties of glass-ceramic scaffolds. Upon immersion in SBF, Fu *et al.* [105] observed a nanostructured hydroxyapatite layer formed on the surface of the porous scaffolds within 7 days (Figure 3), indicating the *in vitro* bioactivity of the scaffolds. Such HA nanocrystals are found in human bone and believed to be beneficial for increased cell adhesion, proliferation and greater tissue growth into the scaffold [84,133,134]. Cell culture results and scanning electron microscopy (SEM) observations presented in ref. [105] confirmed an excellent attachment and subsequent proliferation of MC3T3-E1 pre-osteoblastic cells, both on the surface and in the interior of the scaffolds (Figure 4).

**Figure 5:**
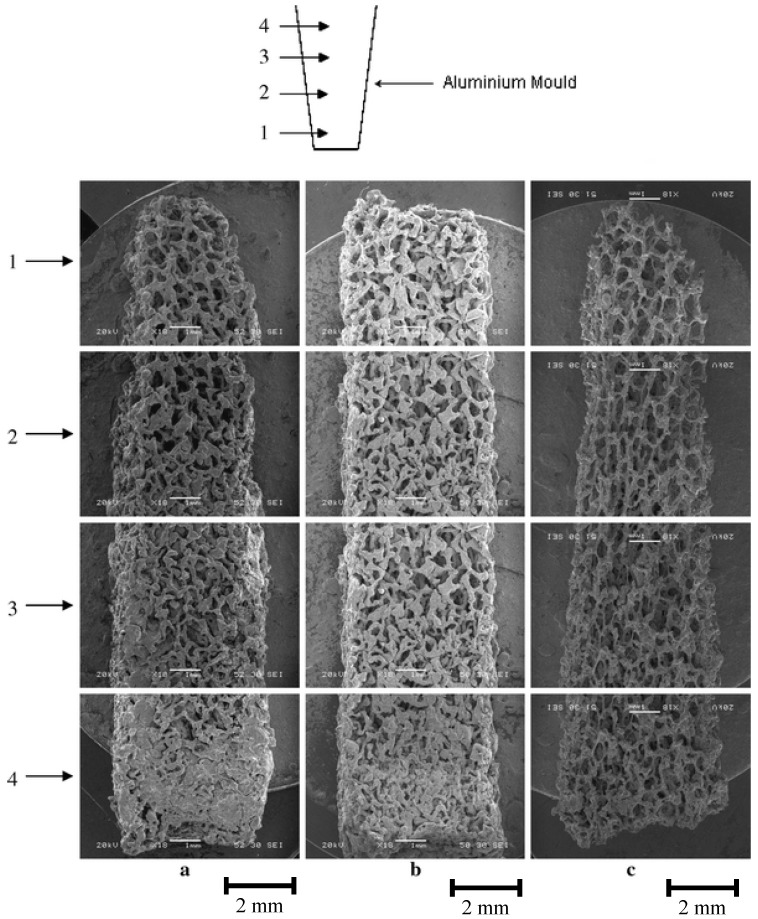
SEM images of scaffolds with 3D continuous porosity gradient after sintering at different degrees of compaction in aluminum mould: (a) 75%, (b) 65%, (c) 50%. Reprinted from ref. [103], with permission from Springer Netherlands.

**Figure 6:**
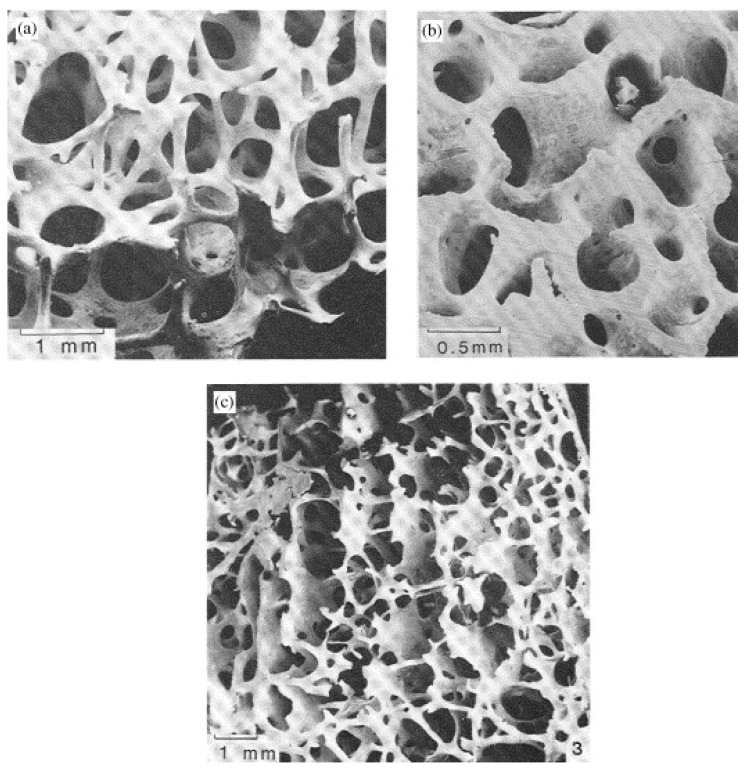
Scanning electron micrographs showing the cellular structure of trabecular bone. (a) Specimen taken from the femoral head, showing low-density, open-cell, rod-like structure. (b) Specimen taken from the femoral head, showing a higher density, perforated plate-like structure. (c) Specimen taken from the femoral condyle, of intermediate density, showing an oriented structure, with rods normal to parallel plates. Figure reprinted from reference [138] with permission of Elsevier.

Another interesting approach in the development of bone TE scaffolds is to engineer constructs with graded porosity. Vitale-Brovarone *et al.* [104] and Bretcanu *et al.* [103] manufactured highly porous bioactive glass-ceramic scaffolds with tailored porosity gradient (Figure 5) in order to mimic the morphology and lightweight structure of human bone, formed by cortical (compact bone with dense structure) and cancellous bone (trabecular bone with highly porous structure). Trabecular bone represents only about 20 wt % of the skeletal mass, but has a nearly ten times greater surface-to-volume ratio (100−300 cm^2^/cm^3^) than compact bone [135,136]. Therefore, trabecular bone is far more important in phosphate and calcium homeostasis than compact bone. The unique hierarchical structure of bone enables its self-repairing properties; bone can alter its geometry (Figure 6) and material properties in response to changing external load stimuli, and it undergoes a continuous remodeling process [132,137]. Bone grows in response to load so that the density of trabecular bone depends on the magnitude of the loads and the orientation of the trabeculae depends on the loading direction (Figure 6). Low-density trabecular bone resembles open-cell foam while high-density trabecular bone has a more plate-like structure, with perforations through the plates [138].

**Figure 7:**
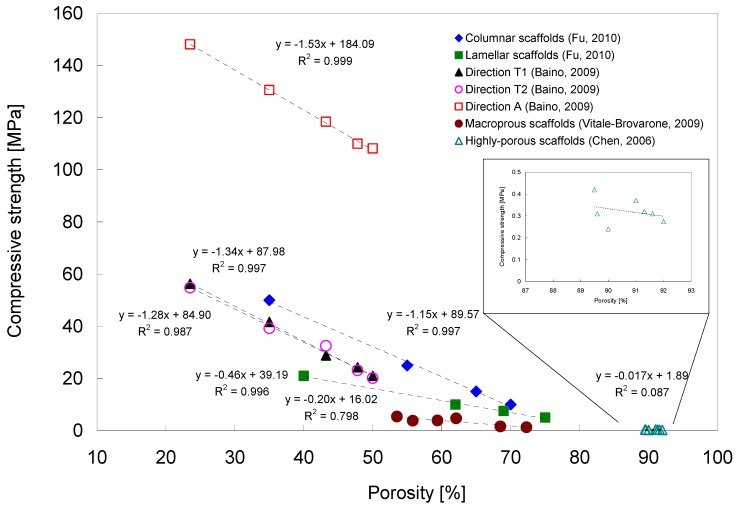
Relationship between porosity and compressive strength of bioactive glass-ceramic scaffolds. Data adapted from refs. [43,94,99,102].

Analyzing experimental results from the literature [43,94,99,102], a highly negative linear relationship between scaffold porosity and compressive strength was found, with coefficients of determination R^2^ between 0.80 and 0.99 (Figure 7), obtained from linear curve fitting. This means that for a particular scaffold, at least 80% of the variability of the compressive strength can be explained by the systematic influence of porosity. Coefficients of determination found for quadratic or exponential functions were in the same order of magnitude. For the different scaffolds, an increase in porosity by 10% has been shown to decrease the compressive strength by 2–15 MPa, variations being represented by the different slopes in Figure 7. The linear relationship failed to hold for very high porosities of 89–92% [43] (Figure 7), which can be explained by the onset of instability phenomena (e.g., buckling) which occur in particular at high porosities and promote the collapse of the scaffold micro-architecture.

The large variations in compressive strength values of the scaffolds can be interpreted by different fabrication methods, glass compositions, pore morphologies, pore sizes, pore size distributions, shape and thickness of struts (leading to anisotropic mechanical properties), as well as by different compressive strength test parameters employed (sample geometry, size, loading speed). A linear correlation has also been found between porosity and elastic modulus of glass-ceramic scaffolds using ultrasonic wave propagation [112] (Figure 9).

**Figure 8:**
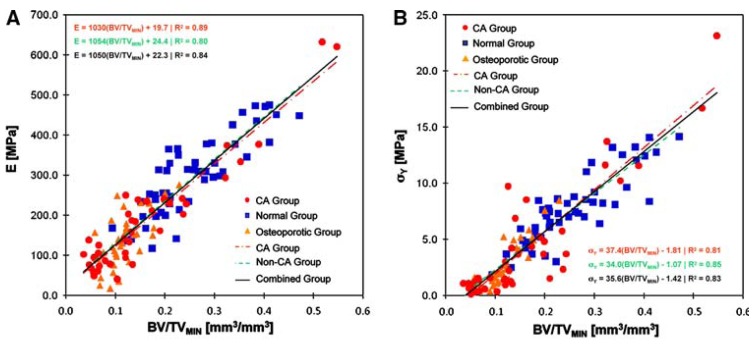
Linear regression models illustrating that (A) compressive modulus of elasticity and (B) compressive yield strength of non-cancer (Non-CA), normal and osteoporotic, and metastatic cancer (CA) cancellous bone specimens are functions of bone volume fraction (BV/TV_MIN_) regardless of the underlying pathology. Specimen groups: cancer (CA, red), non-cancer (green), cancer and non-cancer combined (black). Reprinted from reference [128], with permission of Springer-Verlag, New York.

**Figure 9:**
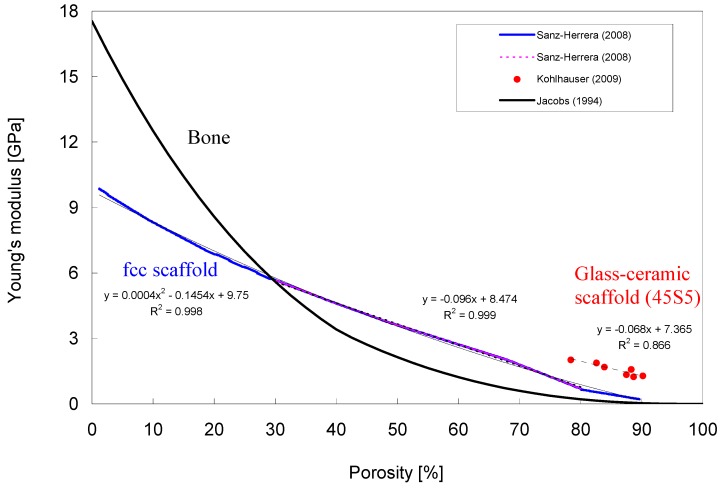
Young’s modulus of an fcc scaffold microstructure [141] and of bone [139] as a function of porosity (Data kindly provided by Prof. J. A. Sanz-Herrera, University of Seville, Spain). The pink, dashed line, overlapping the blue line, represents the data range (porosity of fcc structure = 30–80%), in which a highly linear relationship between porosity and Young’s modulus exists. A second order polynomial function describes the Young’s modulus of the fcc structure over the entire range of investigated porosities (1.2–89.6%). For Bioglass^®^-based glass-ceramic scaffolds (data adapted from ref. [112]), a highly linear negative correlation (coefficient of determination R^2^ = 0.866) between porosity and elastic modulus was found on the basis of ultrasonic wave propagation measurements.

For human bone, different functional relationships between bone volume fraction (*i.e.*, porosity) and mechanical properties have been observed. On the basis of image-guided failure assessment (IGFA), Nazarian *et al.* [128] found highly positive linear correlations (R^2^ = 0.8−0.9) between bone volume fraction and compressive yield strength (Figure 8 B), as well as between bone volume fraction and elastic modulus (Figure 8 A). Other authors reported quadratic [138] or power-law relationships between bone volume fraction (relative density) and compressive strength [125], as well between bone volume fraction and Young’s modulus of human bone (Figure 9) [138,139]. Moreover, a second order polynomial relationship between porosity and Young’s modulus has been found in the modeling of the mechanical properties of a face-cubic-centered (fcc) scaffold microstructure [140,141] (Figure 9). However, the Young’s modulus of fcc microstructures with moderate porosities of between 30% and 80% (pink, dashed overlapped data line in Figure 9) was well-estimated by linear regression analysis.

**Figure 10:**
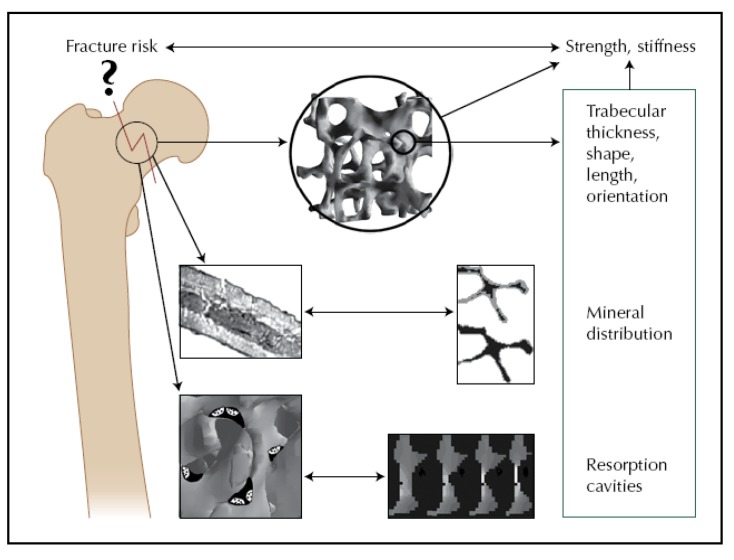
Basal studies investigating cortical and cancellous bone have resulted in information on the effect of trabecular architecture, mineral distribution, and bone remodeling on bone strength and stiffness. As the resolution of clinical imaging techniques and data extraction techniques improve, this information will gradually become available directly from clinical data. Reprinted from ref. [156], with permission of Springer.

The non-linear relationships between porosity and stiffness (E-Modulus) [125,138,139,141] are well in agreement with the homogenization of heterogeneous materials and micromechanics theory of porous solids [138,142], whose details are far beyond the scope of this review. Briefly, the stiffness of a cellular solid depends mainly on its microstructure and the mechanical properties of the base material. This means that the biomechanics of porous solids is determined by a complex interplay between porosity, microstructure and bulk mechanical properties. On the one hand, the porosity determines the resultant microstructure morphology, and consequently a certain degree of anisotropy may eventually occur, in cases where pores show a preferential direction. On the other hand, the intrinsic base material properties (e.g., material composition in the case of scaffolds, or mineral content in bone; see Figure 10) induce the overall stiffness. According to the micromechanics theory, individual curves of different base materials showing the ratio E/E_0_ (being E the apparent Young’s modulus and E_0_ the Young’s modulus of the solid) as a function of porosity match each other if the microstructure (pore/cell geometry of different porosities) is geometrically similar (*i.e.*, no architecture-elastic modulus dependence) and as long as the mode of deformation or failure is the same [138].

Although strength and stiffness underlie completely different physical mechanisms (strength is related to a critical point of material collapse, whereas Young’s modulus is related to the linear elastic relationship between stress and strain), the similarity in the density dependencies of Young’s modulus and compressive strength of human bone, reported as being both linear [128] (see Figure 8) and both quadratic [138], suggest that the failure strain is a constant in cases where material instabilities are not predominant during collapse.

The state of knowledge regarding the relationship between porosity and pore size of biomaterials used for bone regeneration, and the effect of these morphological features on osteogenesis, have been reviewed in detail by Karageorgiou and Kaplan [74] whilst the mechanical properties of bioactive glass-ceramic scaffolds have been summarized by Thompson and Hench [70,98].

In the complex process of bone regeneration, scaffold microstructure and microstructure anisotropy play important roles because both morphological features determine the spatial distribution of the newly formed tissue [143]. The sophisticated hierarchical micro-structure of bone with highly interconnected trabeculae ensures waste removal, nutrient/oxygen supply and protein transport during tissue regeneration and bone growth [95,144]. Because studies have shown that cell growth into a scaffold depends on how well nutrients can permeate through the porous structure [145,146], the permeability of scaffolds, a property directly related to the degree of pore interconnectivity, is a key factor influencing the scaffolds ability to enhance bone tissue regeneration [95]. Permeability quantifies the ability of a porous medium to transmit fluid through its interconnected pores or channels when subjected to pressure, and therefore controls the nutrient flow to cells that migrate through the scaffolds [95].

To our knowledge, there are only a few studies on the permeability assessment/evaluation of porous bioceramic scaffolds including both numerical modeling [147] and experimental determination of permeability constants [95,145,148,149]. In a recent study on bioactive glass-ceramic scaffolds (porosity: 90–95%), Ochoa *et al*. [95] using deionized water as working fluid measured an average permeability constant of 1.96 × 10^−9^ m^2^. This value is close to reported experimental data for bovine (k = 2 × 10^−9^–9.5 × 10^−9^ m^2^, [150]) and human trabecular bone (k = 5.1 × 10^−9^–7.2 × 10^−9^ m^2^, [151]), confirming that the fabricated scaffolds had transport properties as well as pore structure close to trabecular bone.

In this context, it has to be pointed out that the intrinsic permeability is a function of pore morphology (e.g., interconnection, shape and size of pores), as well as overall porosity [95]. This fact implies that scaffolds of the same porosity might have different values in the intrinsic permeability due to differences of the microstructural design of the pore structure and morphology. However, for scaffolds showing a regular microstructure over a wide range of porosities, the permeability has shown to be proportional to the third power of porosity [141]. However, with increasing porosity, the apparent scaffold stiffness and strength decrease [141] (Figure 7, Figure 9). Another point of interest related to the intrinsic scaffold permeability is the attachment and migration of cells to the scaffold surface. This mechanism seems to be dependent on both the bulk biomaterial stiffness [152] and the available specific surface area. The specific surface is not directly related to the permeability although it is influenced by permeability since the specific surface is a function of the micro-structural design of the scaffold and porosity, which determine the overall permeability [95]. As we have discussed above, with higher porosities the permeability of scaffolds increases, whereas the stiffness and specific surface area decrease. Therefore, depending on the particular application, a compromise has to be made in scaffold porosity design.

For human bone, the microstructure–property relationship as well as the relative importance of bone mineral density and bone architecture in the etiology of fractures have poorly been understood and largely unexplored [128,153,154,155,156]. Functional micro-imaging at the interface of mechanics and biology (IGFA) is increasingly becoming a powerful technique to gain insights into the fracture mechanisms of bone. So far, studies have shown that bone strength and stiffness depend strongly on bone mass, but they also depend on the morphometry and micro-architecture of cortical and cancellous bone (e.g., shape, thickness, spacing of trabeculae). All these aspects differ between individuals and between anatomic sites [156]. For completeness of the treatment of this topic in this review, Figure 10 shows influencing factors which determine the strength and fracture risk of human bone.

In addition to providing excellent *in vitro* bioactivity [14,43,94,109,110,115], cell biology behavior [15,105,114,118,119] and favorable mechanical properties [99,102,105], bioactive glass-ceramic scaffolds have shown superior *in vivo* behavior (e.g., bone formation, mineralization, higher interfacial strength between implant and bone) compared to the glass in particulate form [113] or compared to other bioactive materials (HA, tricalcium phosphate) [52].

**Figure 11:**
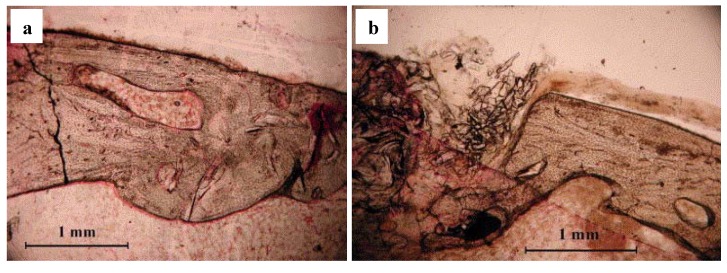
Optical micrograph from histological sections of tibial bone defects after 6 months of implantation of bioactive glass based materials: (a) The histological section of a defect filled with glass fiber scaffold (porosity of 55–60%) shows regenerated cortical bone in the form of mature bony lamellar structure. A bone regeneration lacuna can be identified in the centre of the section and it appears to be free from glass remnants. (b) The histological section of a defect filled with PerioGlas^®^ (particles) shows mature cortical bone with non-homogenous structure. Some regeneration lacunae with fragments of glass are also present. Reprinted from ref. [113] with permission from Elsevier.

In a pilot study, Moimas *et al.* [113] created cortical holes in the tibia of rabbits and filled the cavities with bioactive glass fibrous scaffolds as well as with 45S5 Bioglass^®^ particles (PerioGlas^®^). After 6 months of implantation, the histology showed that three-dimensional implants were more effective than PerioGlas^®^ particles in helping new bone formation and remodeling (Figure 11). Tomographic analysis of the negative control (empty defects) and of the filled defects provided evidence of the superiority of the empty defect as regards cortical bone formation. However, filling using the three-dimensional constructs aided trabecular bone formation also in areas in which bone was not naturally present. In a similar *in vivo* study using a rabbit calvarial bone model, San Miguel *et al.* [124] reported superior osteoconductive behavior (*i.e.*, significantly higher bone formation, bone deposition) of SBF-pretreated scaffolds (BG fiber constructs) compared with non-treated porous BG scaffolds, bioactive glass granules (PerioGlas^®^) and empty bone defects.

### 3.2. Bioactive Glass containing Composite Scaffolds

As discussed in detail above, depending on bioactive glass composition, fabrication method and porosity, a large range of compressive strength values has been reported (0.3–150 MPa) (Figure 7). There is a clear tendency towards lower mechanical strength with increasing porosity, so that a compromise between porosity and mechanical strength has to be made for the particular bone TE application. Most of the prepared glass-ceramic scaffolds in the presented studies reviewed here exhibited a suitable interconnected macroporous network and yielded compression strengths > 2 MPa, being in the range of the compression strength of cancellous bone (Table 2, Figure 7). They therefore can match the criterion in terms of compressive strength, but load bearing bone defect sites are usually under cyclic loading and as the scaffolds are made from porous glass they are normally inherently brittle and have poor tensile strength (Table 2).

**Figure 12:**
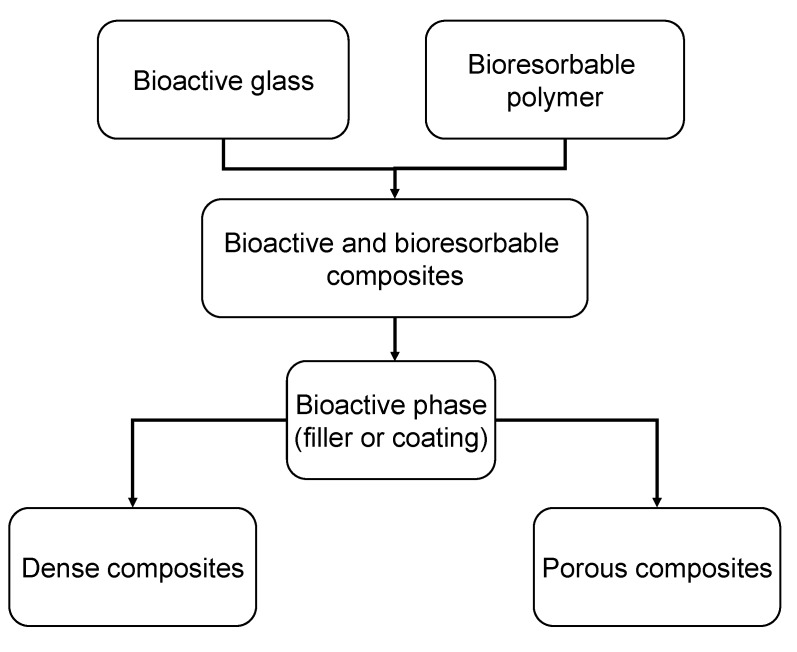
Schematic diagram showing the types of synthetic bioactive and biodegradable polymer composite scaffolds for bone tissue engineering applications (modified after reference [81]).

Fracture toughness values in the range reported for cortical bone (2–12 MPa·m^1/2^) are required for load-bearing applications [70] and therefore toughness must be introduced into this type of scaffolds, which can be achieved by producing composites [10]. Polymer/bioceramic composite scaffolds represent a convenient alternative due to the possibility to tailor their various properties (e.g., mechanical and structural behavior, degradation kinetics and bioactivity) [157]. Composites made of polymers and bioceramics combine the advantages of their singular components [98]. Polymers exhibit generally high ductility, toughness, favorable formability as well as processibility and plasticity. The glass or glass-ceramic phase adds stiffness and adequate mechanical strength to the composite. In particular, composites based on biodegradable polymers are being increasingly studied as bone TE materials because this particular combination does not require a revision surgery for their removal as newly formed bone gradually substitutes the implanted scaffold during degradation [42,81]. However, perhaps the most clinically successful and commercially available bioactive composite on the market is non biodegradable, e.g., it is based on HA and polyethylene [8,158]. Much current research is therefore focused on the fabrication of bioactive composite materials, as both solid and porous systems with the bioactive phase incorporated either as filler or coating (or both) into the bioresorbable polymer matrix (Figure 12, Table 3). Effort is devoted in particular to the development of porous, high-strength composite structures for the regeneration of human bone at load-bearing sites. A comprehensive general review on bone TE scaffolds based on composites with inorganic bioactive fillers has been published by Rezwan *et al.* [37]. The state of knowledge on polymer-bioceramic composites with focus on polymer coatings and interpenetrating polymer-bioceramic structures for bone TE has recently been summarized by Yunos *et al.* [77].

In 2003, Boccaccini and Maquet [42] reported for the first time on the successful fabrication of porous foam-like bioactive glass containing poly(lactide-co-glycolide) (PLGA) composites, which exhibited well-defined, oriented and interconnected porosity. Since then, many studies have been carried out to optimize and investigate bone TE composite scaffolds concerning material combinations, bioactive properties, degradation characteristics [161,164,165,168], *in vitro* [47,161,162,163,169] and *in vivo* behavior [47,164], as well as mechanical properties [50,81,160].

**Figure 13:**
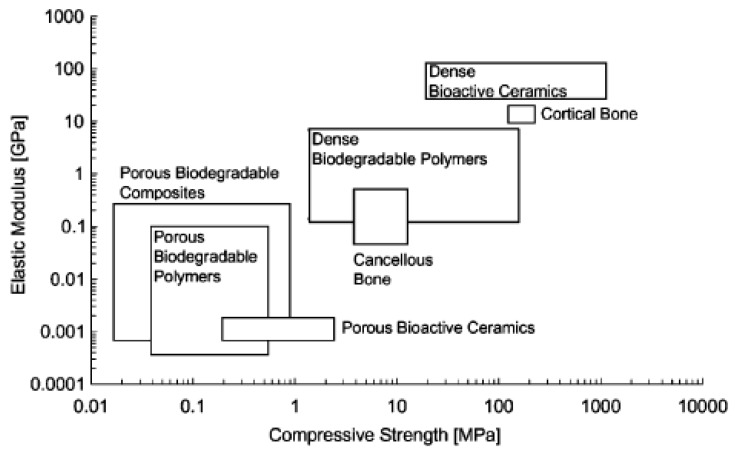
Elastic modulus vs. compressive strength values of biodegradable polymers, bioactive ceramics and composites after ref. [37]. Porosities of scaffolds are considered to be >75% and mostly interconnected. Dense polymers can match cancellous bone properties and approach cortical bone properties. Moreover, the bioactive ceramics region is close to cortical bone. Porous scaffolds, however, are at least one order of magnitude weaker than cancellous bone and orders of magnitude weaker than cortical bone. Figure reproduced with permission of Elsevier.

In particular, with regard to their mechanical properties in comparison to human bone, porous polymer/ceramic (glass) composites scaffolds have revealed insufficient mechanical integrity [37]. So far, the mechanical strength of most of today’s available porous polymer/BG composite scaffolds is inadequate for bone substitution because they are at least one order of magnitude weaker than natural cancellous bone and orders of magnitude weaker than cortical bone (Figure 13). Moreover there is still limited understanding on how microstructure features (e.g., geometry of struts, pore size distribution, pore orientation, interconnectivity, morphology and distribution of the BG filler) affect the scaffold mechanical response and its functional performance [143]. In addition, insufficient particle-matrix bonding is considered a possible reason for the low mechanical properties of these composites. With regard to the latter, two key issues have to be solved to effectively improve the material properties of matrices by adding bioactive glass particles as filler: interfacial bonding and the proper, homogeneous dispersion of the individual particles in the matrix (e.g., by particle surface functionalization). According to the concepts of the composites theory [176], load transfer at the filler (*i.e.*, BG particles)/matrix interface is key to achieve strengthening and stiffening, which depends on the quality of interfacial bonding between the two phases (filler and matrix). Strong interfacial bonding is therefore a significant condition for improving the mechanical properties of biodegradable polymer composite scaffolds containing BG fillers.

**Table 3:** Overview on studies performed on BG containing composite scaffolds for bone TE. Key: PDLLA, poly(D, L lactide); P(3HB), poly3-hydroxybutyrate), P(CL/DLLA), poly(ε-caprolactone/D, L lactide); PLGA, poly(lactic-co-glycolic acid); PDLG, poly(D, L lactide-co-glycolide); PLA, poly(L lactide); S53P4, 53 wt % SiO_2_, 23 wt % Na_2_O, 20 wt % CaO, 4 wt % P_2_O_5_; m-BG, micron-sized bioactive glass; n-BG, nano-sized bioactive glass. ST/PL, sugar template/particulate leaching; TIPS, thermally induced phase separation.

Bioactive glass	wt %	Particle size	Matrix	Fabrication technique/process	Ref.
45S5 m-BG	5, 29, 40	< 40 μm	PDLLA	Co-extrusion+compaction; TIPS	[159]
45S5 m-BG	4.8, 28.6	5–20 μm	PDLLA	TIPS	[160]
45S5 m-BG	10	< 5 μm	P(3HB)	ST/PL	[47]
45S5 n-BG	10	30 nm	P(3HB)	ST/PL	[47]
S53P4 m-BG	20, 50	90–315 µm	P(CL/DLLA)	ST/PL	[161]
S53P4 m-BG	30	< 45 μm	P(CL/DLLA)	ST/PL	[162]
45S5 m-BG	10, 30	< 40 μm	PLGA	Microsphere emulsification	[163]
45S5 m-BG	10	4 μm	PDLG	TIPS	[164]
45S5 m-BG	25, 50	50–63 µm	PLA	Freeze extraction technique	[165]
45S5 m-BG	5, 40	> 90 μm	PDLLA	Solvent casting	[166]
45S5 m-BG	10, 25, 50	< 5 μm	PDLLA	TIPS	[167]
45S5 m-BG	10, 25, 50	< 5 μm	PLGA	TIPS	[167]
45S5 m-BG	5, 10, 40	< 5 μm	PDLLA	TIPS	[168]
45S5 m-BG	5, 40	< 5 μm	PDLLA	TIPS	[169]
45S5 m-BG	10, 25, 50	< 5 μm	PLGA	TIPS	[42]
45S5 m-BG	25	< 40 μm	PLGA	Solvent casting	[50]
45S5 m-BG	20	< 10 μm	P(3HB)	Solvent casting	[170]
45S5 m-BG	20	< 5 μm	P(3HB)	Solvent casting	[171]
45S5 n-BG	10, 20	29 nm	P(3HB)	Solvent casting	[46]
45S5 m-BG	10, 20, 30	< 5 μm	P(3HB)	Solvent casting	[48]
45S5 n-BG	10, 20, 30	30–50 nm	P(3HB)	Solvent casting	[48]
45S5 m-BG	5, 30	5 μm	PDLLA	TIPS	[172]
45S5 m-BG	5, 30	5 μm	PDLLA	TIPS	[173]
45S5 m-BG	5, 40	< 5 μm	PDLLA	TIPS	[174]
SiO_2_-3CaO-P_2_O_5_-MgO	10, 30, 50	10 μm	PLA	TIPS	[175]

**Figure 14:**
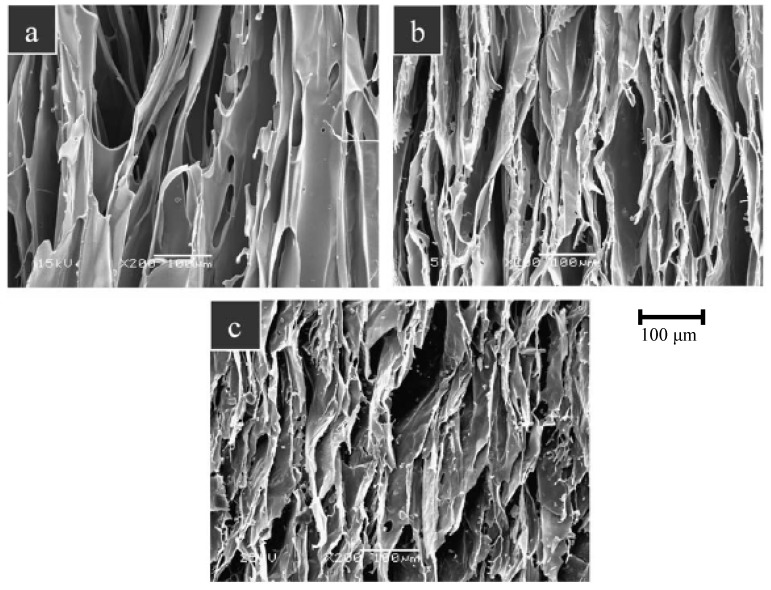
SEM images of a PDLLA/Bioglass^®^ scaffold fabricated by TIPS showing the typical homogeneous, tubular regions of **(a)** pure PDLLA foam, **(b)** PDLLA / 4.8 wt % Bioglass^®^ foam, **(c)** PDLLA / 28.6 wt % Bioglass^®^ foam [160]. Figure reprinted with permission of Elsevier.

Blaker *et al.* [160] have developed highly porous (porosity ≈ 94%) poly(D, L lactide) (PDLLA)/Bioglass^®^ foams using thermally induced phase separation (TIPS). The scaffolds exhibited a bimodal and anisotropic pore structure (Figure 14), with tubular micropores of ≈ 100 µm in diameter, and with interconnected micro-pores of ≈ 50–10 μm, along with anisotropic mechanical properties. With respect to the direction of the tubular pores, similar axial yield strengths of about 0.08 MPa were found for all composites (0, 4.8, 28.6 wt % Bioglass^®^), whereas a higher axial compressive modulus (1.2 MPa) was obtained for 28.6 wt % Bioglass^®^ containing scaffolds compared to the pure PDLLA constructs (0.89 MPa). These yield strength values reported in ref. [160] are considerably lower than those for cancellous bone (yield strength: 5.7–356 MPa [127,128]), so that a further improvement is necessary to increase the mechanical performance towards the levels required for bone TE applications. The compressive moduli are in the range of those determined for trabecular bone, but lower than those for cortical bone (see Table 2.)

Other authors found, however, considerably higher mechanical strength for their composite scaffolds [50,167]. Maquet *et al.* [167], for example, have reported highly porous (porosity > 90%) PDLLA and PLGA scaffolds, containing 50 wt % Bioglass^®^ exhibiting compressive moduli of about 21 MPa and 26 MPa, respectively, being a factor of 1.5–2.5 higher than those for the pure polymer scaffolds. Lu *et al.* [50] determined for PLGA scaffolds incorporated with 25 wt % Bioglass^®^ (porosity = 43%, pore diameter = 89 µm) a compressive modulus of about 51 MPa, and compressive strength of about 0.42 MPa, which is in the range of values reported for trabecular bone (Table 2), however at the cost of porosity (43%).

Interestingly, numerical analyses presented in ref. [160] showed that the compressive modulus of the composite foams can be well predicted by micromechanic theories based on the combination of the Ishai-Cohen [177] and Gibson-Ashby models [178]. The modulus-density (volume fraction) relationship was characterized by a power-law function with exponents between 2 and 3. This is close to the exponents found for similar relationships valid for human bone (2–3.2) [138,139], and to that of an ideal isotropic open-cell porous structure with pores in cubic arrays, which is characterized by an exponent of two [138,178]. On the basis of cellular solids models, this finding suggests that for the porous polymer/BG scaffolds prepared by TIPS [160], struts bending is the dominant mode of linear elastic deformation similar to the trabeculae bending proposed for cancellous bone [138].

**Figure 15:**
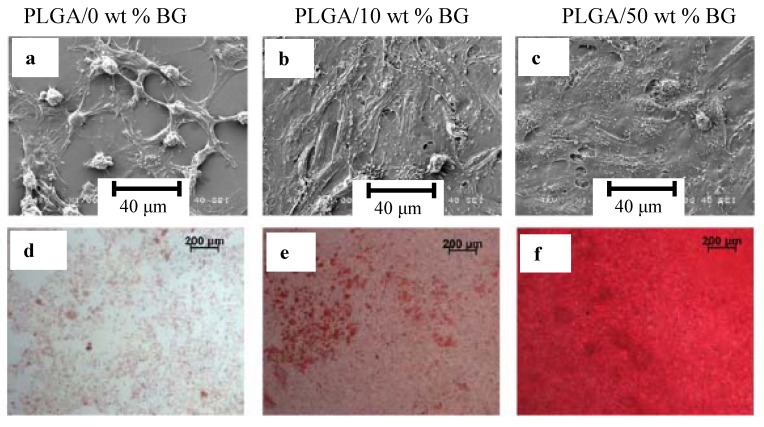
Effect of composition on the growth of osteoblast-like (human osteosarcoma SaOS-2) cells on bioactive glass-PLGA composites (a–c). SEM image in (a) shows lower amount of cells on the pure PLGA surface compared to the two bioactive glass containing composite films (b, c). Alizarin red staining after 28 days in culture (d-f) indicated the positive effect of bioactive glass content on matrix mineralization. The amount of mineralization was significantly higher on the 10 wt % bioactive glass composite (e), likely a combination of cell-mediated mineralization and surface Ca–P formation on the PLGA-bioactive glass composite. Mineralization on sample surfaces: (d) 0 wt %, (e) 10 wt %, (f) 50 wt % bioactive glass containing PLGA composites. Images adapted from Lu *et al.* [179]. Figure published with permission of Elsevier.

Extensive work has been also carried out to investigate the cellular response to bioactive glass containing composites concerning composition, particle concentration and particle size effect *in vitro* and *in vivo* [46,47,48,161,162,163,164,166,169,170,171,174]. For example, Lu *et al.* [179] showed that for PLGA/bioactive glass films (0, 10, 25, 50 wt %), the growth, mineralization and differentiation of human osteoblast-like SaOS-2 cells (Figure 15), as well as the kinetics of Ca–P layer formation and the resulting Ca–P chemistry were dependent on BG content. The 10 wt % and 25 wt % BG composite supported greater osteoblast growth and differentiation compared to the 50 wt % BG group.

Such bioactive glass dose-dependent cell proliferation and ALP synthesis were also reported by Yang *et al.* [174], Verrier *et al.* [169] and Tsigkou *et al.* [166]. Tsigkou *et al.* [166] observed that human fetal osteoblasts were less spread and elongated on PDLLA and PDLLA/5 wt % BG, whereas cells on PDLLA/40 wt % BG were elongated but with multiple protrusions spreading over the BG particles. However, when differentiation and maturation of fetal osteoblasts were examined, incorporation of 45S5 Bioglass^®^ particles within the PDLLA matrix was found to significantly enhance ALP and osteocalcin protein synthesis compared to PDLLA alone. Alizarin red staining indicated extracellular matrix mineralization on 5 wt % and 40 wt % BG containing films, with significantly more bone nodules formed than on neat PDLLA films. Yang *et al.* [174] pre-treated 45S5 BG containing (0, 5, 40 wt %) PDLLA scaffolds with serum and found in human bone marrow mesenchymal stem cells a significant increase in ALP activity in 5 wt % Bioglass^®^ composites relative to the 0 and 40 wt % Bioglass^®^ groups, whereas *in vivo* studies indicated significant new bone formation throughout all the scaffolds. The results of these studies [166,169,174,179] confirmed the osteogenic potential of BG containing scaffolds and suggest that for composites there is a critical threshold range of BG content (5–40 wt %) which is optimal for osteoblast growth and Ca–P formation. This finding might have also consequences for the vascularization and angiogenic properties of composite scaffolds, as discussed in section §4.

To our knowledge, Misra *et al.* [48] were the first who incorporated bioactive glass nanoparticles (30–50 nm) of composition matching the 45S5 BG composition  into degradable matrices (in their case P(3HB) was used) and compared their thermal, mechanical, microstructural, bioactive and cell biological properties with those of conventional, micron-sized BG (5 µm) containing composites. The addition of bioactive glass nanoparticles (n-BG) enhanced the Young’s modulus by 50–100% to values of 1.2 and 1.6 GPa, compared to both pure polymer film and the corresponding micro-sized BG (m-BG) containing films (10, 20, 30 wt %). The nanostructured surface topography induced by n-BG considerably improved protein adsorption on the n-BG composites compared to the unfilled polymer and the m-BG composites, whereas no substantial differences in the proliferation of MG-63 osteoblasts were observed between the different surfaces. The results of this investigation confirmed that the addition of nanosized bioactive glass particles had a more significant effect on the mechanical and structural properties of a composite system in comparison with microparticles, as well as enhancing protein adsorption, two desirable effects for the application of composites in bone tissue engineering.

Tailoring porosity (e.g., nano or mesoporosity [64,180]) and surface topography, e.g., by the incorporation of nanophase bioactive glass particles into degradable polymer matrices, can favor protein adsorption and cellular interactions [48,171], as well as improve the bioactive behavior [46,170], antimicrobial/antibacterial [181,182,183] and mechanical properties [48] of bioactive glass and related (composite) scaffolds. For example, relatively high mechanical properties (compressive strength, Young’s modulus) have been found for polymer matrices incorporating surface functionalized BG nanoparticles prepared from sol-gel routes [49,180]. A further literature overview on sol-gel bioactive glasses and an analysis of functionalization approaches for BG nanoparticles for development of biocomposite materials is however beyond the scope of the present review. Recent key papers on this topic [184,185,186,187,188,189,190] and an informative review highlighting the potential of the sol–gel technology in the research field of bioactive materials for biomedical applications can be found in the literature [34].

## 4. Ion Release from Silicate Scaffolds: Effects on Osteogenesis and Angiogenesis

### 4.1. Ion Dissolution from Bioactive Glasses: Genetic Control of Osteoblast Cell Cycle and Osteogenesis

For many years, it was assumed that formation of a biologically active HCA surface reaction layer was the critical requirement for bioactive behavior [5,8,11]. Today, the formation of a surface HCA layer is considered to be a useful but not the critical stage of reaction for bone regeneration. Key mechanisms leading to enhanced new bone growth are now known to be related to the controlled release of ionic dissolution products from the degrading bioactive glass, especially critical concentrations of biologically active, soluble silica and calcium ions [8,191]. Recent studies have shown that bioactive (partially) resorbable glasses and their ionic dissolution products enhance osteo-genesis by regulating osteoblast proliferation, differentiation, and gene expression [7,41,51,191,192,193,194,195,196,197,198].

**Figure 16:**
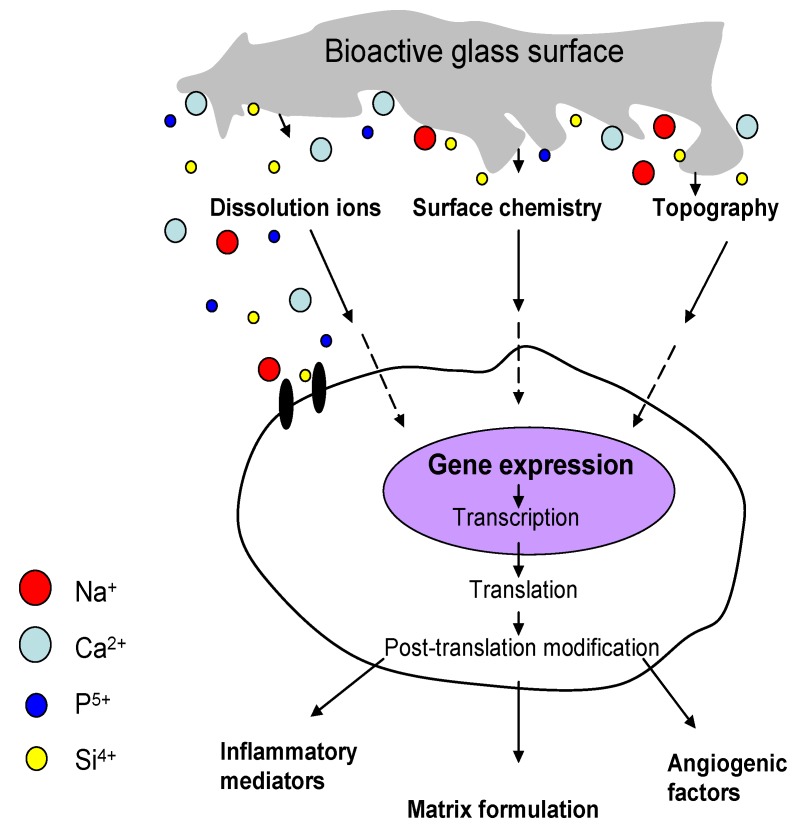
Genetic expression mechanisms in osteoblasts provoked by ion dissolution products of bioactive glasses (adapted from reference [198]).

Bioactive glass is thus proposed to determine bone cell gene expression by four main mechanisms: (1) surface chemistry, (2) topography, (3) rate and type of dissolution ions released and (4) shear stress at scaffold/bone interfaces (mechanical properties) [198] (Figure 16).

Gene activation by controlled ion release provides the conceptual basis for the molecular design of so-called third generation biomaterials [1], which are optimized for *in situ* tissue regeneration. In the case of silicate bioactive glasses, the mechanism for *in situ* tissue regeneration involves up-regulation of seven families of genes that control the osteoblast cell cycle, mitosis and differentiation giving rise to rapid bone regeneration [7,51,191,198]. In order to regenerate bone, it is essential for osteoprogenitor cells to undergo cell division (mitosis) and to receive the correct chemical stimuli from their local environment that instruct them to enter the active segments of the cell cycle [8,41]. Sun *et al.* [196] showed that 45S5 Bioglass^®^ promotes human osteoblast proliferation by reducing the growth cycle to pass through G_1_ and S phase and then enter G_2_ quickly. In the presence of critical concentrations of Si and Ca ions, within 48 h osteoblasts that are capable of differentiating into a mature osteocyte phenotype begin to proliferate and regenerate new bone. Moreover osteoblasts that are not in the correct phase of the cell cycle and unable to proceed towards differentiation are switched into apoptosis by the ionic dissolution products [36,196].

The release rate and therapeutic levels of ions, which are both determined by concentration and particle size of BG (in case of composite materials) or by scaffold morphology and porosity, as well as the relative contribution of specific ion dissolution products from bioactive glasses or Si-substituted calcium phosphates [199] to osteogenesis and angiogenesis have been controversially debated in the literature [53,54]. It has been hypothesized, but to the authors’ knowledge not proven as yet, that the high silicon concentration from bioactive glass could be a major factor in stimulating osteoblasts to grow fast, which might be effective for melt-derived bioactive glasses [7,36,196]. However, Bielby *et al.* [192] found no significant differences in the proliferation of human primary osteoblasts grown in conditioned cell culture media containing similar Ca, P, and Na ions but different Si ion concentrations (164 and 203 ppm) released from 58S sol-gel derived glass. Clearly, more fundamental investigations and further studies are required to gain quantitative knowledge and to confirm the conditions and the mechanisms leading to glass degradation and ion dissolution products affecting gene expression in bone cells. Recent findings also indicate that controlled release of low concentrations of ionic dissolution products from bioactive glasses can induce angiogenesis (as discussed below: §4.3 and 4.4). Thus understanding the role of ions released from bioactive glasses in given concentrations and release rates will lead to the design of gene activating glasses [7,41,51,193] and bioactive glass (composite) scaffolds with osteogenic and angiogenic properties [53,56] offering increased potential for the regeneration of complex tissue structure defects at soft-hard tissue interfaces (e.g., tendon-bone interface).

### 4.2. The Role of Angiogenesis in Bone Regeneration

The lack of a functional microvasculature connected to the host blood supply has been identified as the culprit for implant failure and is currently acknowledged as the major challenge in tissue engineering [200]. Bone is a highly vascularized tissue reliant on the close spatial and temporal connection between blood vessels and bone cells to maintain skeletal integrity. Angiogenesis (or neo-vascularization) plays therefore a key role in skeletal development and bone regeneration [100]. However, unlike organ transplants where there is a preexisting vascular supply, man-made bone TE scaffolds are devoid of vasculature [100]. In addition to the development of pre-vascularized scaffolds *in vitro*, one particular approach being suggested in the field of bioactive glass scaffolds is the controlled release of gene activating ions from bioactive glasses that could promote angiogenesis and bone morphogenesis *in vivo* [53,100]. Because recent studies have shown that the combination of angiogenic and osteogenic factors can stimulate bone healing and regeneration [201,202], the design of advanced bone TE scaffolds with controlled composition of bioactive glass and scaffold microstructure, as well as with controlled local ion release kinetics from biodegradable materials is considered a promising strategy to enhance the repair mechanism of critical sized bone defects. The role of angiogenic and osteogenic factors in the adaptive response and interaction of osteoblasts and endothelial cells during the processes of bone development and bone repair has been highlighted in a review by Kanczler and Oreffo [100]. While a further analysis of the cell biology and *in vivo* aspects of this topic is beyond the scope of the present review, the next section summarizes the state of the art in the field of angiogenic effects of bioactive glasses, a topic of increasing research interest [47].

### 4.3. Effect of Bioactive Glass on Angiogenesis

A detailed overview on studies investigating bioactive glasses with respect to angiogenesis has been recently published by Gorustovich *et al.* [53]. Cell culture studies demonstrated the pro-angiogenic potential of BG over a limited range of BG concentrations implying that dose-dependent effects are also involved in angiogenesis similar to those shown for osteogenic differentiation (ALP synthesis) and cell behavior (adhesion, growth; see §3.2) [166,169,174,179]. Experiments have shown that bioactive glass stimulates the secretion of angiogenic growth factors in fibroblasts [54,164,203,204,205], the proliferation of endothelial cells [29,54,56,204,206], and the formation of endothelial tubules [54,56], as discussed next.

Human fibroblasts in direct contact with 45S5 Bioglass^®^ coatings (0.0625, 0.3125, 0.625 mg/cm^2^, particle size < 5 μm) have shown to secrete significantly higher vascular endothelial growth factor (VEGF) compared to uncoated surfaces [54]. Similar results were found for human microvascular endothelial cells attached to BG coatings (≈0.06, 0.6 mg/cm^2^) [56]. In their study, Leu *et al.* have shown that BG has a biphasic nature in that it possesses proangiogenic potential over a limited range of concentrations and greater osteogenic potential at higher concentrations [56].

In the case of BG-filled composites, filler weight percentages of 0.0625, 0.625 and 6.25 wt % [55], as well as of 1 and 10 wt % [164] have shown angiogenic stimuli in human CCD-18Co fibroblasts and mouse (L929) fibroblasts, respectively. Day *et al.* [55] found that L929 fibroblasts cultured on the surface of PLGA/Bioglass^®^ discs with 0.01%, 0.1%, and 1% (w/v) 45S5 Bioglass^®^ particles (size < 5 μm), equivalent to 0.0625–6.25 wt %, secreted increased amounts of VEGF compared with cells cultured on PLGA alone. In a related study, Keshaw *et al.* [164] recently reported that microporous spheres of PLGA containing 10 wt % 45S5 Bioglass^®^ particles (mean particle size = 4 µm) stimulated a significant increase in VEGF secretion from CCD-18Co fibroblasts consistently over a 10-day period compared with neat PLGA microporous spheres. Moreover, murine preosteoblastic cells (MC3T3-E1) cultured on porous 3D PLGA scaffolds have shown enhanced angiogenic expression (VEGF secretion, VEGF expression) in comparison to cells cultured on two-dimensional PLGA films [207]. A “dimension response element” has been suggested to be involved in the regulation of osteogenic and angiogenic gene expression [207], which supports the hypothesis formulated above that the geometry and morphology of the scaffold are important factors controlling the mechanisms of angiogenesis.

**Figure 17:**
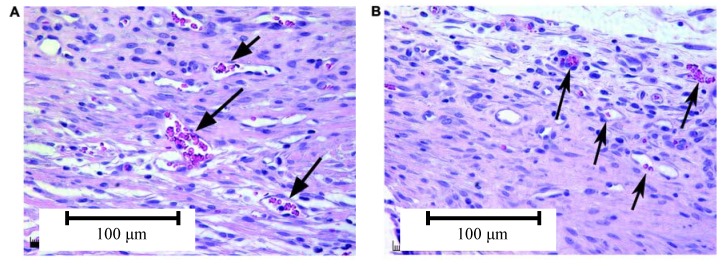
Two-week decalcified tissues stained with hematoxylin/eosin and treated with BG-loaded collagen sponge (A) or collagen control (B) Arrows denote blood vessels. Reprinted from ref. [211] with permission of Mary Ann Liebert.

*In vivo* results have confirmed that BG is able to stimulate and promote neo-vascularization [52,55,120,121,122,164,203,206,208,209,210,211]. For example, Leu *et al*. [211] filled calvarial defects in Sprague-Dawley rats with 45S5 Bioglass^®^ impregnated (1.2 mg) collagen sponges (volume = 0.05 cm^3^) and unloaded, empty sponge as a control. After two weeks of implantation, histological analyses of calvaria demonstrated significantly greater neo-vascularisation and vascular density within defects treated with 45S5 BG (35 ± 16 vessels/mm^2^) than with collagen controls alone (12 ± 2 vessels/mm^2^) (Figure 17).

The angiogenic effect of bioactive glass was, however, much more pronounced in bioactive glass-based scaffolds (*i.e.*, loaded sponges [56], discs [208], meshes [203], tubes [209] and porous glass-ceramics scaffolds [52,121,122]) than in composite structures incorporating and fully embedding bioactive glass particles (e.g., microsphere composites [164], or foams [55,210]). However, *in vivo* results so far are inconsistent with *in vitro* results, and provide an incomplete picture concerning the suggested angiogenic potential.

Keshaw *et al.* [164] reported that microporous spheres of PLGA containing 10 wt % 45S5 Bioglass^®^ particles stimulated *in vitro* a prolonged and significant increase in VEGF compared to pure polymer constructs but observed *in vivo* for the same scaffolds no significant difference in the number of blood vessels infiltrating the voids between microporous spheres. The authors concluded that the presence of well-vascularized voids inside the neat PLGA microporous spheres suggests either that the inclusion of an angiogenic stimulus is not necessary to promote scaffold neo-vascularization at the implant site, or that the normal wound healing response has masked the angiogenic stimulus initiated by bioactive glass.

From the few studies which investigated the angiogenic potential of BG-filled composites with filler contents of 0.625 wt % [55], 10 wt % [164], and 30 wt % [210], only Day *et al.* [55] found favorable angiogenic properties (*i.e.*, greater tissue infiltration and higher blood vessel formation) for compression-molded BG composites compared to the corresponding unfilled polymer scaffolds. Interestingly, the same authors found no difference in the number of formed blood vessels for scaffolds prepared by TIPS technology. This result indicates that the geometry and morphology (pore orientation, pore size, interconnectivity) of the scaffold affect the angiogenic response of the construct *in vivo* [53,54,55].

## 5. Conclusions and Future Work

One of the most significant challenges in bone tissue engineering remains the fabrication of scaffolds exhibiting mechanical, structural, surface-chemical, topographical and biological properties suitable to regenerate large (critical size) cortical bone defects and capable of functioning under relevant loads. Although a number of bioactive glass and glass-ceramic scaffolds with favorable properties are available as comprehensively discussed in this review, several issues need to be addressed prior to clinical application, such as mechanical reliability of scaffolds, tailored degradability, and induction of vascularization. In bone TE, the major challenge remains the proper cellularization and controlled vascularization of 3D scaffolds. For successful bone regeneration, there is a need for functional, mature vessels promoting functionality to the intrinsically “inactive” man-made TE constructs. In angiogenesis, the development of mature blood vessels is necessary because these have the ability to differentiate into arteries and veins. One alternative to accelerate osteogenesis and angiogenesis is the incorporation of active biomolecules such as growth factors into the scaffold structure [206,212,213,214,215,216]. However, short half-life and uncontrolled release of growth factors from scaffolds associated with possible toxicity effects are a problem or limitation of current drug delivery scaffolds. The use of bioactive glass as filler in degradable matrices might offer a promising strategy for the regulated *in situ* secretion/expression of angiogenic growth factors (e.g., VEGF) and osteogenic markers (e.g., ALP) in therapeutic levels leading to successful vascularization and bone formation (mineralization) of TE scaffolds.

Further improvement of scaffold function is related to surface modification, e.g., through the control of specific/non-specific protein adsorption [217], plasma treatment [218,219] or enzyme grafting [220], to provide biofunctional groups for cell attachment and response, thus making the scaffold more surface compatible. There is still limited understanding regarding the long-term *in vivo* behavior of porous 3D silicate scaffolds and polymer/BG composite scaffolds, particularly regarding the degradation, ion release kinetics and angiogenic stimulus of these highly porous systems. In this context, it has to be pointed out that the influence of sterilization on the cytotoxic, mechanical (e.g., compressive strength, fracture toughness) and physical properties (glass transition temperature, crystallinity) of biodegradable composites has often been overlooked in the past. This is particularly important for scaffolds incorporating a polymeric phase. Sterilization issues have to be considered and monitored in parallel to the design and development stages of the scaffolds because standard medical product sterilization techniques (gamma irradiation, ethylene oxide gas exposure) have shown to reduce molecular weight of resorbable polymers by a factor of 2–3 [221,222,223].

Moreover, more focus on *in vivo* studies is inevitable and there is need for more research on the application of scaffolds in realistic biological systems. Engineered scaffolds from silicate amorphous or partially crystallized systems, combined with biodegradable polymers, shall continue being improved and optimized. These scaffolds may constitute the “scaffolds of choice” in future developments and their combination with stem cells is of high interest [62,224,225,226]. The use of bioactive glass and glass ceramic nanoparticles [10,44] and carbon nanotubes (CNTs) [89,227,228] as well as their combination with bioresorbable polymers [46,47,48,89,170,229] may also improve the environment to enhance cell attachment, proliferation, angiogenic and osteogenic properties as well as adding extra functionalities to the base scaffold. However, possible toxicity issues associated with nanoparticles and CNTs will have to be comprehensively investigated [89].
